# Effects of dietary glycerol monodecanoate supplementation on inflammation, oxidative stress, ghrelin acylation, and gastrointestinal transcriptome in Yanbian cattle fed a high-concentrate diet

**DOI:** 10.5713/ab.251032

**Published:** 2026-04-02

**Authors:** Xin Feng, Xinyu Liu, Shibin Fang, Yutong Liu, Xiaoxue Yang, Lingzhu Lin, Yun Dai, Tianyu Dong, Xin Du, Jiaming Luan, Chunyin Geng

**Affiliations:** 1 Agricultural College, Yanbian University, Yanji, China; 2 Engineering Research Center of North-East Cold Region Beef Cattle Science & Technology Innovation, Ministry of Education, Yanbian University, Yanji, China

**Keywords:** Ghrelin, Glycerol Monodecanoate, High-concentrate Diet, Inflammation, Oxidative Stress

## Abstract

**Objective:**

This study aimed to investigate the effects and potential mechanisms of dietary glycerol monodecanoate (GMD) supplementation on gastrointestinal inflammation and oxidative stress in Yanbian cattle fed a high-concentrate diet.

**Methods:**

Thirty Yanbian cattle were randomly assigned to two groups: a control group (CON) fed a basal high-concentrate diet, and a treatment group (C10) fed the same basal diet supplemented with 60 g/cattle/day GMD. After a 10-day adaptation period, the experiment lasted 90 days. Collected samples (serum, rumen epithelium, abomasum, ileal mucosa) were analyzed for the following: gastrointestinal morphology (rumen and ileum); inflammatory, antioxidant, and oxidative stress indices (serum, rumen, and ileum); ghrelin-related indices (serum and abomasum); and host transcriptomes (rumen and ileum).

**Results:**

GMD supplementation significantly reduced the levels of tumor necrosis factor-α and interleukin-1β (IL-1β) in serum and rumen, as well as IL-1β in the ileum. It also lowered oxidative stress, as indicated by lower reactive oxygen species in serum, rumen, and ileum. Concurrently, GMD increased antioxidant capacity by increasing levels of reduced glutathione and antioxidant enzymes. Beneficial trends in gastrointestinal morphology were noted in the C10 group, including increased ruminal papillae height and a higher ileal villus height-to-crypt depth ratio. Furthermore, the C10 group exhibited enhanced ghrelin acylation, with increased total ghrelin, acylated ghrelin and ghrelin O-acyltransferase activity in both serum and abomasum. Transcriptomic analysis of the C10 group compared to the CON group revealed tissue-specific protective mechanisms: in the rumen epithelium, pro-inflammatory pathways including NF-κB signaling were significantly suppressed, accompanied by down-regulation of genes such as *IL1B* and *CXCL2*, whereas the ileal mucosal response was dominated by marked up-regulation of *GSTA1*.

**Conclusion:**

GMD supplementation can reduce inflammation and oxidative stress in Yanbian cattle fed a high-concentrate diet. These effects may involve activation of ghrelin acylation and tissue-specific modulation of transcriptional programs in the rumen and ileum.

## INTRODUCTION

High-concentrate (high-grain) feeding is commonly used to increase energy intake and growth in cattle, but it predisposes animals to subacute ruminal acidosis (SARA), which has been associated with reduced feed intake, impaired performance, and increased health risks [1–4]. Under this condition, prolonged ruminal pH depression promotes microbial dysbiosis and elevates free lipopolysaccharide (LPS) in ruminal digesta, leading to LPS translocation and subsequent systemic inflammatory responses [1–3]. In parallel, acidic conditions and endotoxin exposure can compromise epithelial barrier integrity in both the rumen and hindgut, thereby promoting gastrointestinal inflammation [1–3]. Moreover, high-concentrate feeding challenges are frequently accompanied by oxidative stress and compromised antioxidant defenses [4]. As a derivative of medium-chain fatty acids (MCFAs), glycerol monodecanoate (GMD) and its active metabolite, decanoic acid (capric acid), have been demonstrated to exert multiple health-regulating effects in monogastric animals, including improvement of gastrointestinal microbiota, enhancement of antioxidant capacity, and modulation of inflammatory responses [5,6]. In rodent models, the study by Zhang et al [5] showed that GMD increased the abundance of beneficial bacteria in mouse feces in a dose-dependent manner, improved intestinal barrier integrity, thereby reducing LPS translocation, and significantly lowered serum levels of pro-inflammatory cytokines such as interleukin-1 beta (IL-1β) and tumor necrosis factor-alpha (TNF-α). In studies using IPEC-J2 cells and a miniature pig model, Lee and Kang [6] confirmed that decanoic acid could effectively alleviate cyclophosphamide-induced intestinal inflammation, oxidative stress, and barrier damage through multiple mechanisms. However, evidence regarding the mechanisms and efficacy of GMD or decanoic acid in the complex gastrointestinal system of ruminants remains scarce. Previous studies have primarily focused on the effects of decanoic acid on modulating rumen fermentation parameters and inhibiting methane production [7,8].

Ghrelin is a multifunctional gastric peptide hormone produced primarily in the gastric fundus, and its bioactivity mainly depends on the post-translational acylation and modification by ghrelin O-acyltransferase (GOAT) [9]. Importantly, dietary MCFAs can serve as direct substrates for GOAT, promoting the synthesis of acylated ghrelin (AG) [10]. In ruminants, studies have preliminarily revealed that ghrelin secretion is regulated by feeding rhythms and rumen fill, and it is involved in the regulation of growth hormone secretion and energy metabolism [11–13]. A recent study has shown that supplementing with MCFA monoglycerides can alleviate oxidative stress in cattle, with GMD demonstrating the most significant effect [14]. This effect may be related to the activation of the ghrelin system, suggesting that GMD may indirectly exert anti-inflammatory and antioxidant effects by modulating the ghrelin system [14]. Nevertheless, knowledge remains very limited regarding the connection between ghrelin and gastrointestinal health (especially inflammation and oxidative stress) in ruminants.

At present, although MCFAs are recognized for their ability to reshape the rumen microbiota in ruminants and to enhance intestinal barrier function in monogastric animals by reducing ileal inflammation [14–16], the effects of GMD on local tissue responses in the rumen epithelium and ileal mucosa, particularly under high-concentrate dietary stress, remain largely unknown. Therefore, this study evaluated the effects of GMD on inflammatory markers, antioxidant status, and ghrelin concentration in serum and gastrointestinal tissue of Yanbian cattle. Transcriptomic analysis of rumen epithelium and ileal mucosa was additionally integrated to elucidate potential mechanisms, supporting the application of GMD as a functional feed additive for ruminants.

## MATERIALS AND METHODS

This experiment was conducted from June to September 2024 at Longjing Mule Animal Husbandry. GMD (oily solid, purity≥99%) was purchased from Shandong Binzhou Jinsheng New Material Technology.

### Animals, feeding, and experimental design

Thirty Yanbian cattle (bulls, initial body weight [BW]: 503±48 kg) were randomly divided into two groups: (1) the control group (CON) was fed a basal high-concentrate diet, and (2) the experimental group (C10) was fed the same basal high-concentrate diet supplemented with GMD at 60 g/cattle/day (approximately 0.12 g/kg BW based on the mean initial BW of 503 kg). The GMD dose was selected based on a previous *in vitro* rumen fermentation study [8], in which the appropriate MCFA concentration was estimated according to the rumen fluid volume of adult cattle, and was further supported by an *in vivo* study in rumen-cannulated cattle showing beneficial effects on oxidative stress indices and ghrelin-related parameters [14]. To ensure dose consistency, GMD was administered individually. For each bull in the C10 group, the daily dose (60 g) was weighed, thoroughly premixed with 100 g of concentrate, and offered before the morning feeding; for the CON group, an equivalent amount (100 g) of concentrate without GMD was provided in the same manner. Consumption of the offered concentrate (100 g with or without GMD) was visually confirmed for all animals before the remaining basal diet was offered. The experiment lasted for 100 days, including 10 days of adaptation and 90 days of data collection. All bulls were housed in individual pens, fed a total mixed ration (TMR; concentrate: forage ratio of approximately 70:30 on a dry matter basis, with corn straw as the forage source) twice daily (at 05:00 and 15:00). Fresh water was available ad libitum throughout the experiment. The ingredient and nutrient composition of the basal diet (DM basis) is presented in Table 1.

### Sample collection

On the final day of the experiment, blood samples were collected from all cattle via venipuncture using vacuum blood collection tubes. After clotting, the samples were centrifuged at 3,186×g for 20 min at 4°C to obtain serum, which was aliquoted and stored at −80°C for the measurement of ghrelin, inflammatory, antioxidant, and oxidative stress indices.

At the end of the experiment, following a 12-hour fasting period, 20 randomly selected bulls (10 per group) were transported to a commercial slaughterhouse (Yanji City, Jilin Province, China) for slaughter. The rumen epithelium was excised from the ventral sac region, and abomasal tissue was collected from the fundic region. Ileal mucosa was carefully scraped from the mid-segment of the ileum using sterile glass slides, and a portion of the intact ileal tissue was preserved. All collected tissue samples were rinsed with sterile ice-cold phosphate-buffered saline (PBS). Pieces of rumen and ileal tissue (approximately 4 cm^2^) were fixed in 4% paraformaldehyde solution for morphological examination. Additional portions of the tissues were snap-frozen in liquid nitrogen and transferred to −80°C for storage. These preserved samples were intended for the measurement of ghrelin in abomasal tissue, as well as for the analysis of inflammatory markers, antioxidant and oxidative stress indices, and host transcriptome in the rumen epithelium and ileal mucosa.

### Measurement of rumen and ileum epithelial morphology

Tissue samples from the rumen and ileum (n = 20 per tissue) were fixed in 4% paraformaldehyde for at least 48 h and processed by passing through a standard ethanol dehydration series, followed by xylene clearing and paraffin embedding. Sections of 4–5 μm thickness were cut using a rotary microtome and mounted on glass slides. After deparaffinization and rehydration, sections were stained with hematoxylin and eosin (H&E), dehydrated, cleared in xylene, and coverslipped with a synthetic mounting medium for subsequent histological examination. Morphometric measurements were performed to determine rumen papillae height (μm), papillae width (μm), connective tissue width (μm), stratum corneum thickness (μm), and non-keratinized epithelium thickness (μm), as well as ileal villus height (μm), crypt depth (μm), and the villus height to crypt depth ratio as previously described by Alqaisi et al [17] and Belote et al [18]. All images were captured using an Olympus DP73 microscope (Olympus) and the measurements were performed using Digimizer image analysis software (ver. 5.4.4; MedCalc Software).

### Analysis of ghrelin-related indices

Total ghrelin (TG), deacylated ghrelin (DAG), and GOAT activity in serum (n = 30; 15 per group) and abomasum (n = 20, 10 per group) were analyzed using commercial enzyme-linked immunosorbent assay (ELISA) kits (YJ446287, YJ233015, and YJ623074; Shanghai Enzyme-linked Biotechnology) according to the manufacturer’s instructions. Absorbance was measured on a microplate reader (Spark; Tecan). The concentration of AG was calculated as the difference between TG and DAG. Detailed procedures were as described by Luan et al [19].

### Analysis of inflammatory, antioxidant, and oxidative stress indices

The concentrations of TNF-α (ML077389) and IL-1β (ML064295) in serum (n = 30; 15 per group), rumen epithelium, and ileal mucosa (both tissues: n = 20 each, 10 per group) were quantified using ELISA kits (Shanghai Enzyme-linked Biotechnology). All ELISA measurements were performed following the manufacturer’s instructions.

The levels of total antioxidant capacity (TAOC, HY60021), catalase (CAT, HYM0018), superoxide dismutase (SOD, HYM0001), reduced glutathione (GSH, HY60006), oxidized glutathione (GSSG, HY60008), glutathione reductase (GR, ML076441), glutathione S-transferase (GSH-ST, HY50120), glutathione peroxidase (GSH-PX, HYM0004), reactive oxygen species (ROS, HYM0087), and malondialdehyde (MDA, HYM0003) in serum (n = 30; 15 per group), rumen epithelium, and ileal mucosa (both tissues: n = 20 each, 10 per group) were measured using commercial assay kits. All kits were obtained from the Beijing Sino-UK Institute of Biological Technology, except for the GR assay kit, which was sourced from Shanghai Enzyme-linked Biotechnology. The methods used to measure TAOC, CAT, SOD, GSH-PX, ROS, and MDA were as previously described [14,20]. The GSH content was determined via direct reaction with DTNB at 412 nm. The GSSG content was specifically assessed with the same method following the masking of GSH with 2-vinylpyridine and the enzymatic reduction of GSSG by GR. The GSH-ST assay was based on its catalytic activity of conjugating glutathione with 1-chloro-2,4-dinitrobenzene measured at 340 nm.

### Transcriptome analysis of rumen epithelium and ileal mucosa by RNA sequencing

Total RNA from rumen epithelial and ileal mucosal tissues was extracted using TRIzol Reagent (Life Technologies). In total, 24 samples were analyzed, including 6 biological replicates per group (CON and C10) for each tissue. RNA integrity and concentration were assessed using an Agilent 2100 Bioanalyzer (Agilent Technologies) and a NanoDrop 2000 (Thermo Fisher Scientific), respectively. Sequencing libraries were prepared from 1 μg total RNA per sample using the Hieff NGS Ultima Dual-mode mRNA Library Prep Kit for Illumina (Yeasen Biotechnology) and sequenced on an Illumina NovaSeq platform to generate paired-end reads (2×150 bp).

Clean reads were aligned to the Bos taurus reference genome (GCF_002263795.3; ARS-UCD2.0) using HISAT2 (ver. 2.0.4). Transcript assembly and expression quantification were performed using StringTie (ver. 2.2.1), and gene abundance was reported as FPKM values for expression visualization. Differential expression analysis was performed using the DESeq2 package (ver. 1.30.1) based on raw gene count matrices. Genes with an adjusted p-value (false discovery rate, FDR)<0.1 and an absolute log₂ fold change (FC)>0.5 were considered significantly differentially expressed. Volcano plots were generated to display statistical significance (−log10[FDR]) versus log_2_ FC, and heatmaps were constructed based on Z-score normalized expression values across samples using the heatmap package in R, with hierarchical clustering applied to both genes and samples. Functional enrichment analysis of differentially expressed genes (DEGs) was performed against the Kyoto Encyclopedia of Genes and Genomes (KEGG) database using clusterProfiler (ver. 4.4.4), and results were visualized using Sankey diagrams to show the relationships between DEGs and significantly enriched pathways. Gene set enrichment analysis (GSEA) was also conducted using clusterProfiler.

### Analysis of real-time quantitative polymerase chain reaction

Total RNA was isolated from rumen, abomasum, and ileum tissues (n = 20 per tissue; 10 per group) using an RNA extraction kit (DP419; Tiangen), and RNA concentration was measured with a NanoDrop ND-1000 (Thermo Fisher Scientific). First-strand cDNA was synthesized using FastKing gDNA Dispelling RT SuperMix (KR118; Tiangen). Gene-specific primers (listed in Supplement 1) were used for SYBR Green–based quantitative polymerase chain reaction (qPCR) (SuperReal PreMix Plus, FP205; Tiangen) on an Agilent Mx3005P system. Each 20 μL reaction contained 10 μL 2× master mix, 0.6 μL each primer (10 μM), 2 μL cDNA, and nuclease-free water. Cycling conditions were 95°C for 15 min, followed by 40 cycles of 95°C for 10 s, 60°C for 60 s, and 72°C for 32 s. Relative mRNA abundance was calculated using the 2^^−Δ ΔCt^ method. Glyceraldehyde-3-phosphate dehydrogenase (GAPDH) was used as the reference gene for the abomasum, and β-actin (ACTB) was used for the rumen and ileum [21–23].

### Statistics and analysis

Data on rumen and ileum epithelial morphology, ghrelin, inflammatory, antioxidant, oxidative stress indices, and qPCR results were analyzed and visualized using GraphPad Prism (ver. 9.5.0; GraphPad Software). The normality of all data was assessed using the Shapiro-Wilk test. Normally distributed data were analyzed by Student’s t-test, while non-normally distributed data were analyzed by the Mann-Whitney U test. Significance was set at p<0.05, and a trend was declared at 0.05≤p<0.10. Spearman correlation analysis was conducted to explore correlations among variables. The results were presented as a correlation heatmap generated using Origin (ver. 9.8.0, CorrelationPlot; OriginLab).

## RESULTS

### Histomorphology of the rumen and ileum

**Figure 1:**
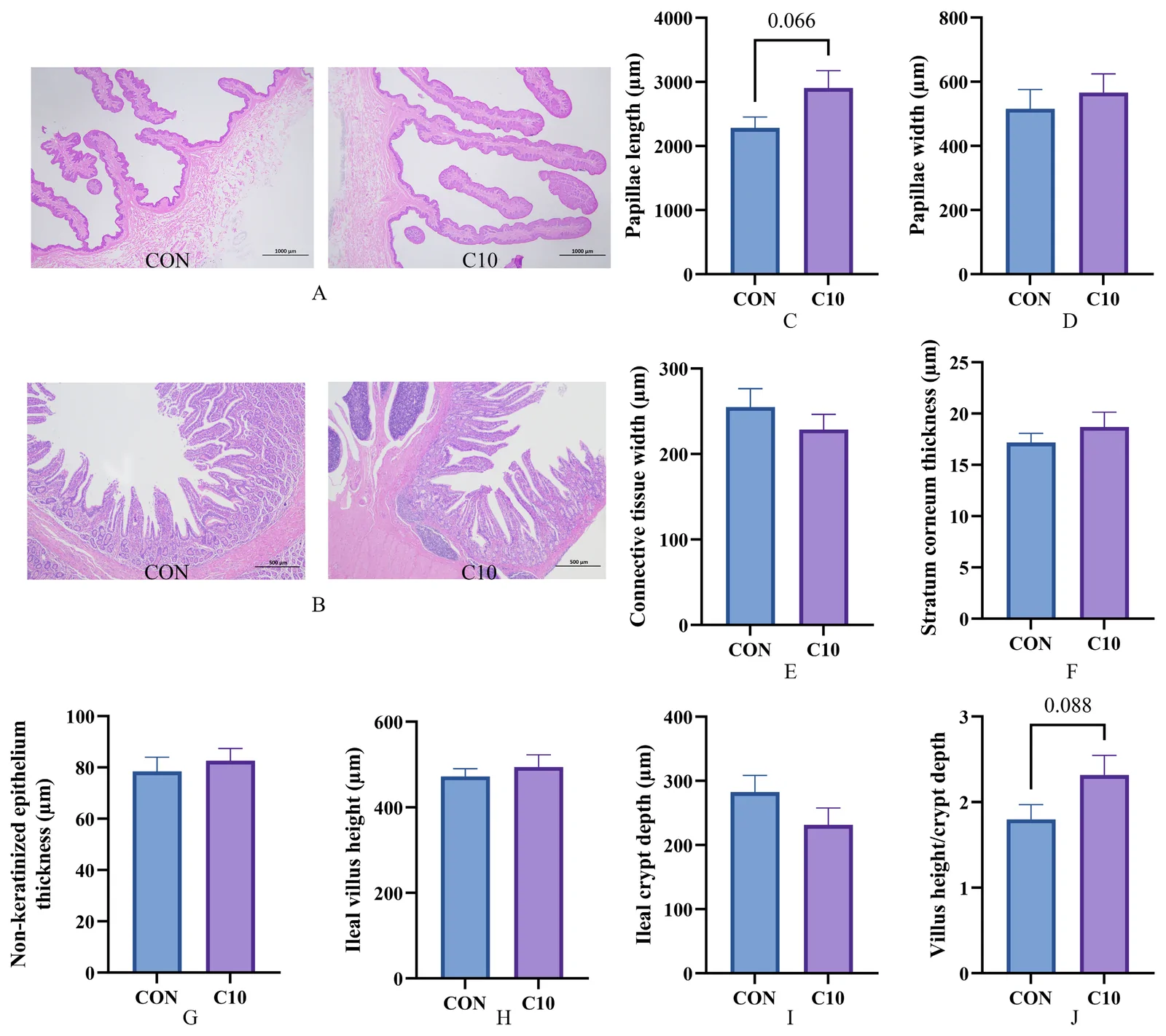
Effects of glycerol monodecanoate (GMD) supplementation on the histomorphology of the rumen and ileum. All sections were stained with hematoxylin and eosin (H&E). Representative photomicrographs of the (A) rumen (×20, 1,000 μm scale bar) and (B) ileum (×40,500 μm scale bar) from CON and C10 groups. Morphometric analysis of the rumen epithelium: (C) papillae height, (D) papillae width, (E) connective tissue width, thickness of the (F) stratum corneum and (G) non-keratinized epithelium. Morphometric analysis of the ileal mucosa: (H) villus height, (I) crypt depth, and (J) villus height to crypt depth ratio. CON: basal high-concentrate diet; C10: basal high-concentrate diet+60 g/cattle/d of GMD.

Histomorphology of the rumen and ileum indicated that dietary supplementation with GMD promoted beneficial morphological changes in both tissues (Figure 1). In the rumen, although most parameters did not reach statistical significance, histological examination revealed noticeably longer and well-developed papillae in the C10 group, which was supported by a trend toward increased papillae height (p = 0.066, Figure 1C). In the ileum, the C10 group exhibited a trend toward an increased villus height to crypt depth ratio (p = 0.088, Figure 1J).

### Inflammatory, antioxidant, and oxidative stress indices

Dietary GMD supplementation significantly ameliorated systemic and gastrointestinal inflammation and oxidative stress in Yanbian cattle. In serum (Table 2), the C10 group exhibited significantly lower concentrations of TNF-α, IL-1β, and ROS, compared to the CON group (p<0.05). Concurrently, the TAOC, CAT activity, GSH concentration and the activities of glutathione-related enzymes (GR, GSH-PX, and GSH-ST) were significantly elevated (p<0.05). SOD activity tended to increase (p = 0.075), whereas MDA tended to decrease (p = 0.092). In the rumen epithelium (Table 3), TNF-α and IL-1β levels were significantly reduced (both p<0.05) in the C10 group. ROS was also significantly decreased (p<0.05), while GSH concentration was significantly increased (p<0.05). In addition, the C10 group showed beneficial trends in TAOC, GR activity, and MDA levels (p = 0.087, 0.099, and 0.072, respectively). In the ileal mucosa (Table 4), supplementing GMD significantly reduced the levels of IL-1β and ROS (p<0.05). Moreover, the GSH concentration and GSH-ST activity significantly increased in the C10 group (p<0.05), and TAOC also showed an upward trend (p = 0.066).

### Ghrelin-related indices in serum and abomasum

**Figure 2:**
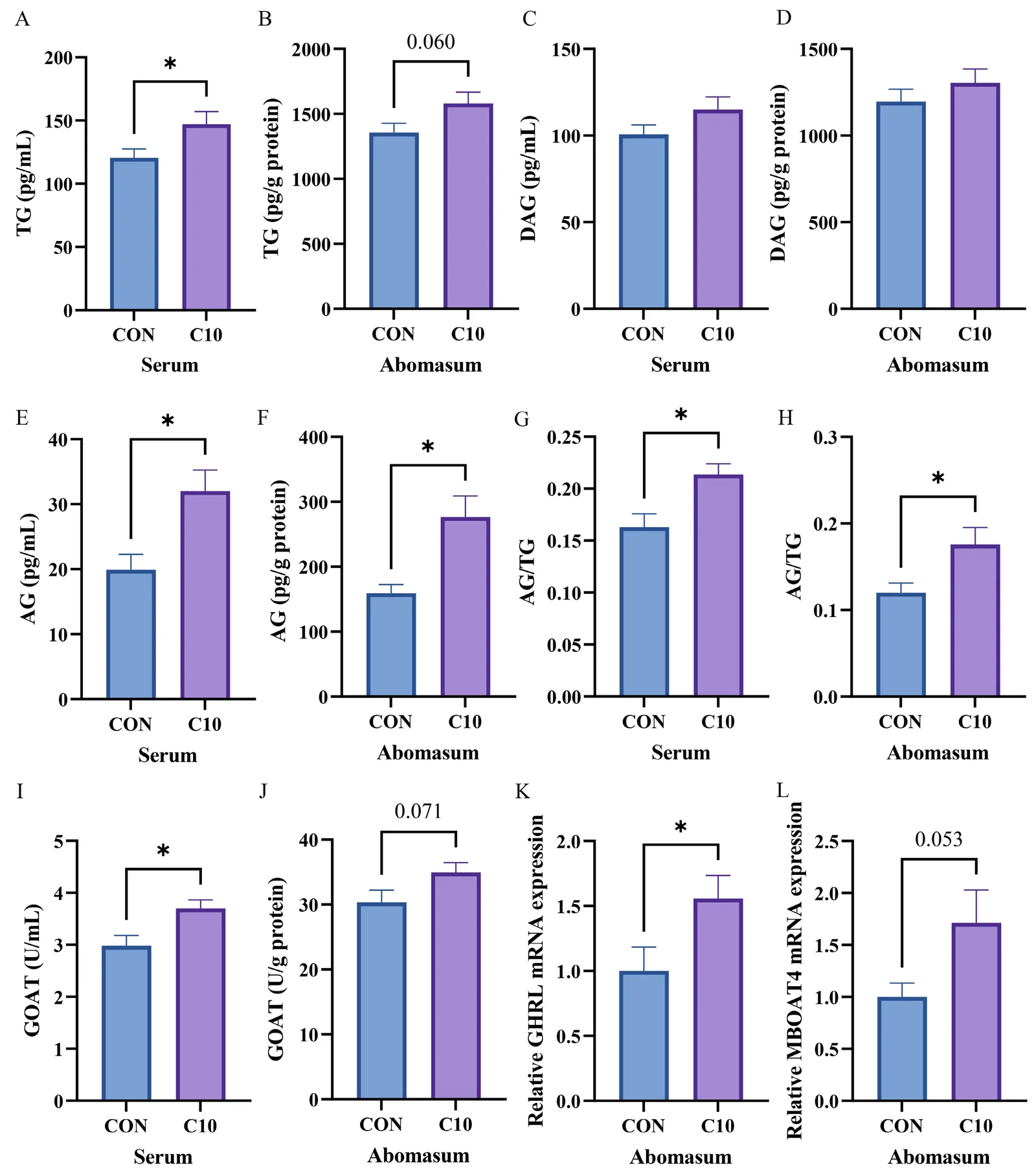
Effects of glycerol monodecanoate (GMD) supplementation on ghrelin-related indices in serum and the abomasum. Concentrations of total ghrelin (TG; A, B), deacylated ghrelin (DAG; C, D), acylated ghrelin (AG; E, F), the AG/TG ratio (G, H), and ghrelin O-acyltransferase (GOAT; I, J) activity were measured in serum (n = 30) and abomasum (n = 20). Relative mRNA expression levels of *GHRL* (ghrelin; K) and *MBOAT4* (GOAT; L) were determined in abomasum (n = 20). Data are presented as mean±SEM. CON: basal high-concentrate diet; C10: basal high-concentrate diet+60 g/cattle/d of GMD. * p<0.05. SEM, standard error of the means.

GMD supplementation significantly influenced ghrelin acylation (Figure 2). In the serum, compared with the CON group, the concentrations of TG and AG were significantly increased in the C10 group, resulting in a significantly higher AG/TG ratio (p<0.05; Figures 2A, 2E, 2G). GOAT activity was also significantly higher in the serum of the C10 group (p<0.05; Figure 2I). A similar activating pattern was observed in the abomasum. While the increase in TG concentration showed a strong trend (p = 0.060; Figure 2B), the levels of AG and the AG/TG ratio were significantly elevated (p<0.05; Figures 2F, 2H). The activity of GOAT in the abomasum also exhibited an increasing tendency (p = 0.071; Figure 2J). At the molecular level, the relative mRNA expression level of *GHRL* was significantly upregulated in the abomasum of the C10 group (p<0.05; Figure 2K). Furthermore, the expression level of *MBOAT4*, which encodes the GOAT enzyme, showed a strong upregulation trend (p = 0.053; Figure 2L).

### Transcriptome analysis

Transcriptomic profiling of 24 samples was performed via eukaryotic reference-based RNA sequencing. In total, we obtained 150.46 Gb of high-quality clean data, with each sample yielding no less than 5.89 Gb, and the percentage of bases with a quality score≥Q30 reaching 94.80% or higher (Supplement 2). To evaluate the sufficiency of sequencing depth for robust gene quantification, we performed a saturation analysis. As shown in Supplement 3, the saturation curves for genes with low to moderate expression levels plateaued at a high value (saturation>0.9) early in the data sampling process, indicating that the current sequencing depth was adequate to reliably detect the majority of expressed genes.

**Figure 3:**
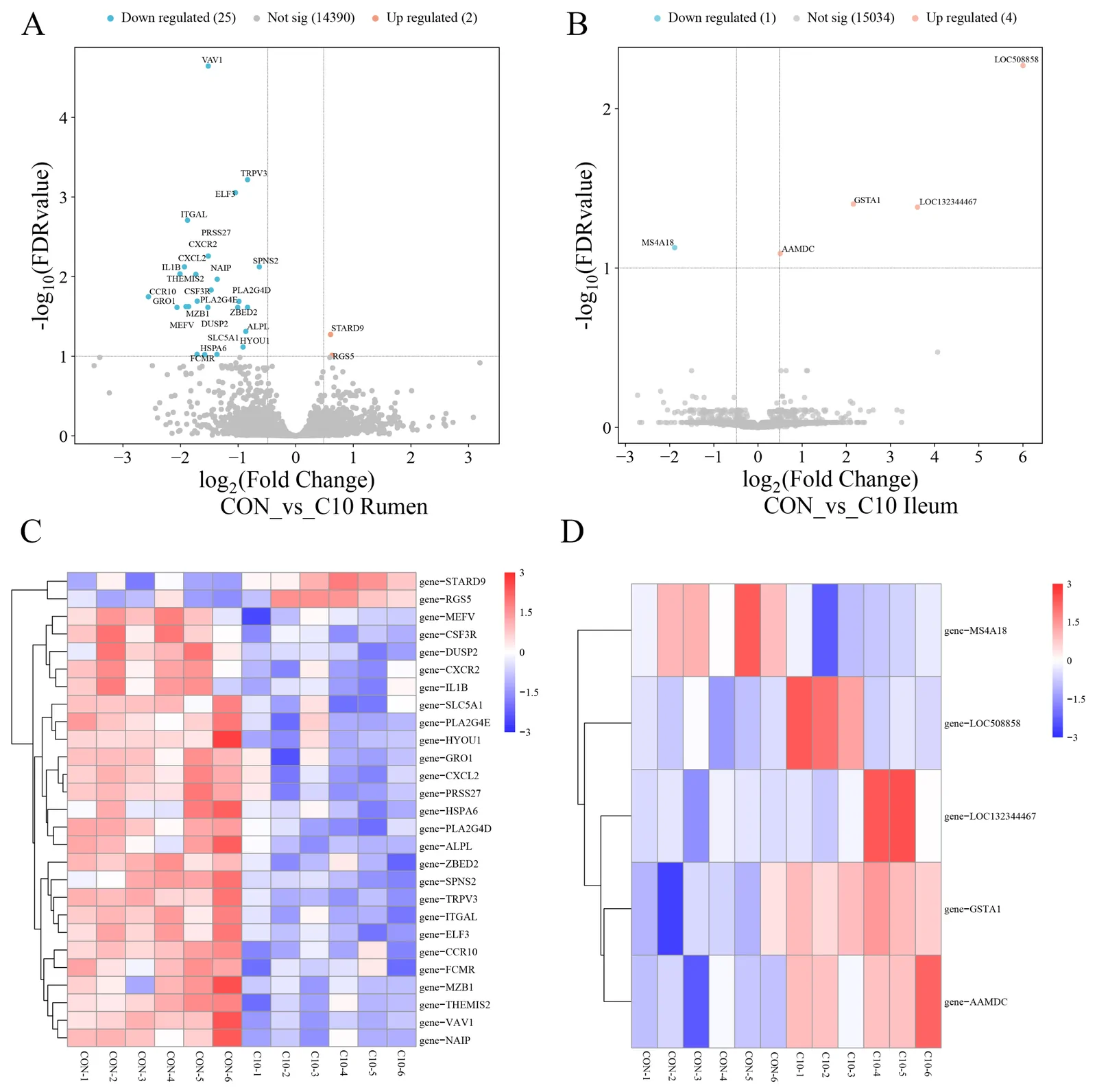
Differentially expressed genes (DEGs) in rumen epithelium and ileal mucosa between CON and C10 (false discovery rate [FDR]<0.1 and |log_2_ fold change [FC]|>0.5). Volcano plots showing DEGs in rumen epithelium (A) and ileal mucosa (B) for the CON vs. C10 comparison. Hierarchical clustering heatmaps of DEGs in rumen (C) and ileum (D). CON, basal high-concentrate diet (n = 6); C10, basal high-concentrate diet supplemented with 60 g/cattle/d glycerol monodecanoate (n = 6).

In the rumen epithelium, 27 DEGs were identified between CON and C10, including 25 down-regulated and 2 up-regulated genes (Figure 3A), whereas the ileal mucosa showed a more limited response with 4 up-regulated and 1 down-regulated gene (Figure 3B). Hierarchical clustering based on these DEGs separated CON and C10 samples into distinct groups in both tissues (Figures 3C, 3D).

**Figure 4:**
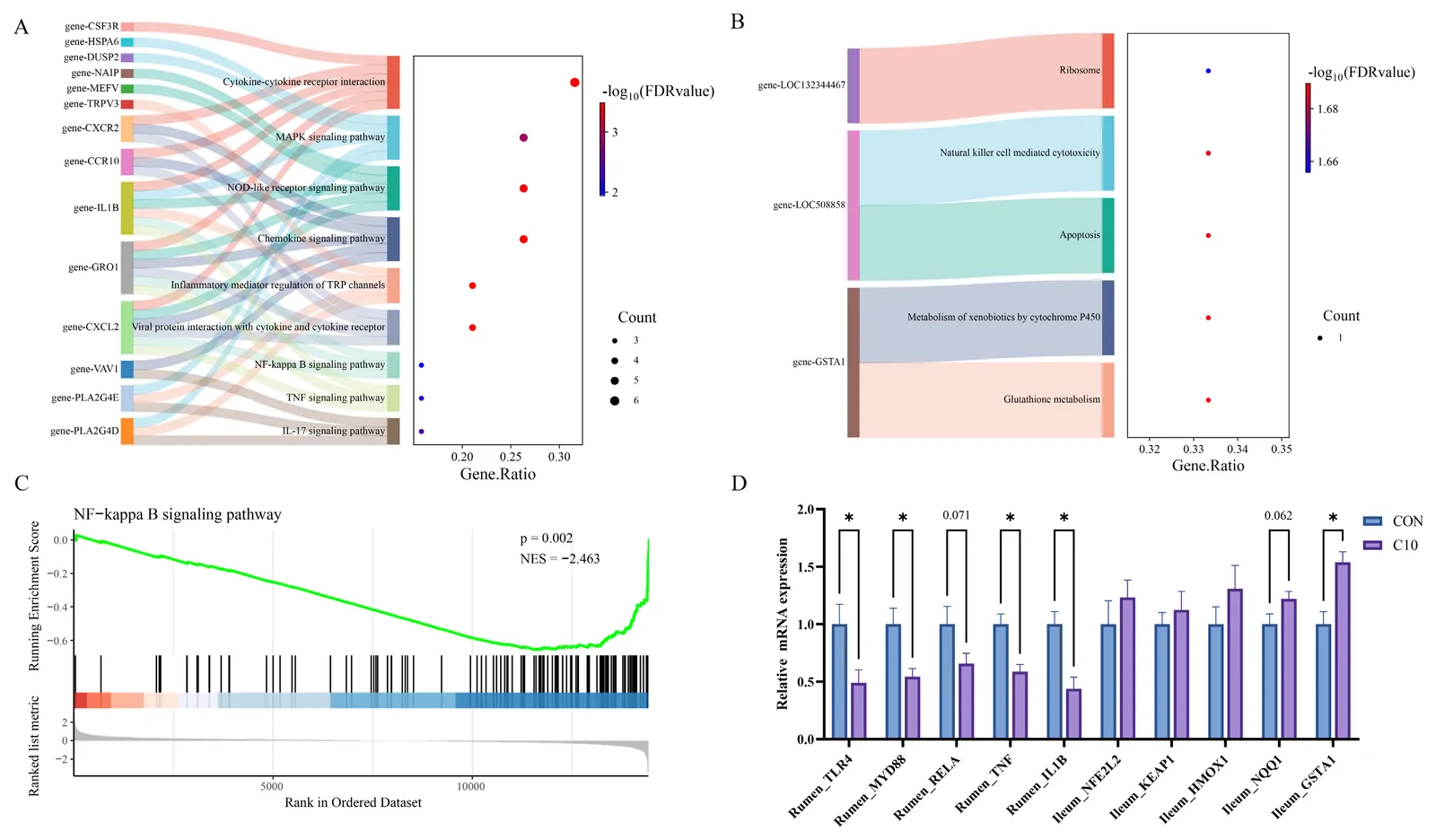
KEGG pathway enrichment, GSEA, and quantitative real-time polymerase chain reaction (qPCR) validation of DEGs in rumen and ileum. Sankey diagrams of KEGG pathway enrichment for DEGs in the rumen (A) and ileum (B). The bubble color represents the enrichment significance (FDR), and the bubble size represents the number of DEGs enriched in the pathway. (C) GSEA enrichment plot of the NF-κB signaling pathway in the rumen. (D) qPCR validation of representative genes in the rumen and ileum (CON vs. C10, n = 10 per group). * p<0.05. KEGG, Kyoto Encyclopedia of Genes and Genomes; GSEA, gene set enrichment analysis; DEGs, differentially expressed genes; FDR, false discovery rate.

In the KEGG enrichment analysis of rumen DEGs, the down-regulated genes mainly participated in immune and inflammatory pathways, such as cytokine-cytokine receptor interaction, chemokine signaling pathway, TNF signaling pathway, IL-17 signaling pathway, NOD-like receptor signaling pathway, MAPK signaling pathway, and NF-κB signaling pathway (Figure 4A). This result was further confirmed by GSEA, which showed significant negative enrichment of NF-κB signaling pathway in the rumen (NES = −2.463, p = 0.002; Figure 4C). GSEA is highly sensitive to consistent expression changes in gene sets. The suppression of this pathway was supported by the down-regulation of key pro-inflammatory genes, including *IL1B* (log_2_FC = −2.01, FDR<0.05), *CXCL2* (log_2_FC = −1.93, FDR<0.05), and *GRO1* (log_2_FC = −1.86, FDR<0.05) (Supplement 4). qPCR validation confirmed significant down-regulation of upstream regulators (*TLR4* and *MYD88*, p<0.05), core component (*RELA*, p = 0.071), and downstream effectors (*TNF* and *IL1B*, p<0.05) in the NF-κB pathway (Figure 4D). In contrast, the ileal mucosa exhibited a distinct transcriptional profile centered on detoxification processes (Figure 4B). Pathway analysis identified significant enrichment in glutathione metabolism and metabolism of xenobiotics by cytochrome P450, primarily attributed to marked up-regulation of GSTA1 (log_2_FC = 2.16, FDR<0.05) (Supplement 4). qPCR confirmed significant up-regulation of *GSTA1* and an increasing trend for *NQO1* (p = 0.062), whereas *NFE2L2*, *KEAP1*, and *HMOX1* were not significantly changed (Figure 4D).

### Correlation analysis

**Figure 5:**
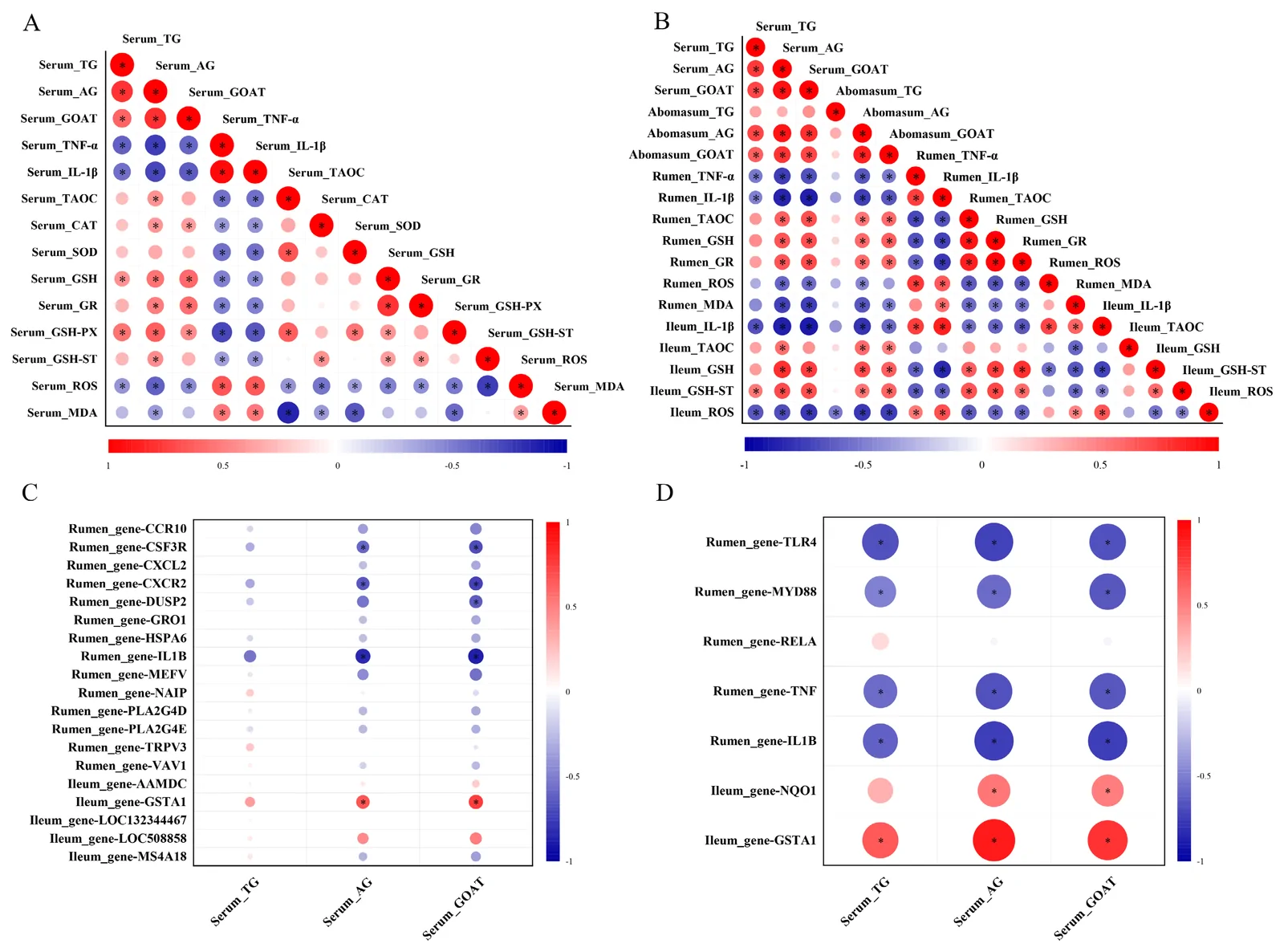
Spearman correlation analysis linking ghrelin indices with inflammatory/antioxidant/oxidative stress indices and gene-expression responses following glycerol monodecanoate (GMD) supplementation. (A) Correlations among serum ghrelin indices (TG, total ghrelin; AG, acylated ghrelin; GOAT, ghrelin O-acyltransferase), inflammatory markers (TNF-α, tumor necrosis factor alpha; IL-1β, interleukin-1 beta), and antioxidant and oxidative stress indices (TAOC, total antioxidant capacity; CAT, catalase; SOD, superoxide dismutase; GSH, reduced glutathione; GR, glutathione reductase; GSH-ST, glutathione S-transferase; GSH-PX, glutathione peroxidase; ROS, reactive oxygen species; MDA, malondialdehyde) (n = 30). (B) Correlations between circulating/abomasal ghrelin indices and local parameters in rumen epithelium and ileal mucosa (n = 20). (C) Correlations between serum ghrelin indices and transcriptome-identified differentially expressed genes (DEGs) in the rumen epithelium and ileal mucosa (n = 12). (D) Correlations between serum ghrelin indices and the expression of key genes validated by quantitative real-time polymerase chain reaction (qPCR) (n = 20). The color and size of the circles represent Spearman’s correlation coefficients. * p<0.05.

Correlation analyses revealed systematic associations between ghrelin-related indicators and key markers of inflammation and redox status across serum, rumen, and ileum tissues. In serum (Figure 5A), TG, AG, and GOAT were each significantly negatively correlated with TNF-α, IL-1β, and ROS (p<0.05), and positively correlated with GSH and GSH-PX (p<0.05). GR and CAT were positively correlated with AG and GOAT (p<0.05). GSH-ST and TAOC were positively correlated with AG (p<0.05). Among the inflammatory, antioxidant, and oxidative stress indices, TNF-α and IL-1β showed significant positive correlations with oxidative stress markers (ROS and MDA) but significant negative correlations with antioxidant indices (p<0.05). In the integrated analysis (Figure 5B), serum and abomasum AG and GOAT were positively inter-correlated (p<0.05). Ghrelin indicators from both serum and abomasum (especially AG and GOAT) showed significant negative correlations with local inflammatory and oxidative stress markers, including rumen TNF-α, rumen IL-1β, rumen MDA, ileal IL-1β, as well as ROS in the rumen and ileum (p<0.05). In contrast, ghrelin indicators (especially AG and GOAT) were positively correlated with antioxidant indices, including rumen TAOC, GSH, and GR, and ileal TAOC, GSH, and GSH-ST (p<0.05).

At the transcript level (Figure 5C), correlation matrices linking serum TG, AG, and GOAT with DEGs showed tissue-specific patterns. In the rumen epithelium, *CSF3R*, *CXCR2*, *DUSP2*, and *IL1B* were significantly negatively correlated with serum AG and serum GOAT (p<0.05). In the ileal mucosa, *GSTA1* was significantly positively correlated with serum AG and serum GOAT (p<0.05). In Figure 5D, serum ghrelin indicators were significantly associated with the qPCR-validated genes. Serum TG, AG, and GOAT were significantly negatively correlated with rumen *TLR4*, *MYD88*, *TNF*, and *IL1B* (p<0.05). In the ileum, *NQO1* was significantly positively correlated with serum AG and serum GOAT, and *GSTA1* was significantly positively correlated with serum TG, AG, and GOAT (p<0.05).

## DISCUSSION

GMD, a derivative of MCFA, has been reported to improve serum antioxidant capacity and alleviate oxidative stress in rumen-cannulated finishing cattle [14], which is consistent with our findings. Importantly, the present study suggests that the antioxidant benefit of GMD is not restricted to the systemic circulation but may extend to the gastrointestinal mucosa. More specifically, TAOC, GSH, GR, and GSH-ST levels were elevated in the rumen epithelium or ileal mucosa, while ROS levels were reduced in both tissues. These results suggest that GMD may improve mucosal redox homeostasis by promoting glutathione-mediated detoxification. Beyond its direct ROS-scavenging role and maintenance of intracellular thiol redox balance, GSH may also exert anti-inflammatory effects by attenuating ROS-driven activation of NF-κB [24,25]. Indeed, inflammation and oxidative stress are mutually reinforcing: ROS can act as signaling molecules that promote NF-κB activation and amplify inflammatory transcription, while the glutathione system helps weaken this oxidative stress–inflammatory positive feedback loop [26,27]. In this study, the reduction in ROS, TNF-α, and IL-1β, coupled with the increase in GSH and antioxidant enzyme activity, suggests that GMD exerts coordinated anti-inflammatory and antioxidant effects in cattle. In addition, GMD tended to exert beneficial effects on gastrointestinal morphology, reflected by greater ruminal papilla height and a higher villus height-to-crypt depth ratio in the ileum. Hanczakowska et al [28] and Chwen et al [29] also reported similar results in piglet studies, finding that decanoic acid or medium-chain triacylglycerols (MCTs) supplementation can promote ileal villus development. Given the overall reduction in inflammation and oxidative stress, the observed morphological improvement likely reflects tissue recovery due to reduced injury.

Another significant finding of this study is the marked activation of ghrelin acylation by GMD, as indicated by increased TG, AG, the AG/TG ratio, and GOAT activity, together with increased or upward-trending expression of *GHRL* and *MBOAT4* in the abomasum. Dietary MCFAs can serve directly as acyl donors for ghrelin acylation. Nishi et al [10] reported that ingestion of MCFAs or MCTs increased gastric AG levels in mice, and that the acyl chain length of newly synthesized ghrelin matched that of the ingested fatty acids. In addition, GOAT has been identified as the key enzyme catalyzing ghrelin acylation, with AG production determined jointly by substrate availability and enzymatic activity [9]. In ruminants, the abomasum is a major site of ghrelin production, and ghrelin mRNA expression in the abomasum is substantially higher than that in other gastrointestinal segments in cattle [30]. GMD supplementation may modulate gastrointestinal microbiota and metabolite profiles, which could potentiate the GOAT-mediated ghrelin acylation process and thereby contribute to elevated circulating AG levels [14]. Importantly, a large body of evidence indicates that ghrelin mediates immunomodulatory and anti-inflammatory effects by suppressing pro-inflammatory cytokine production and inhibiting NF-κB activation in immune and endothelial cells [31,32]. Under lipotoxic or inflammatory stimulation, ghrelin can also inhibit TLR4/NF-κB signaling and alleviate oxidative stress–associated injury [33,34]. Moreover, studies by Wang et al [35] and El-Shaer et al [36] have shown that ghrelin enhances antioxidant defenses through pathways such as Nrf2/HO-1. In line with these mechanisms, this study found that circulating AG and GOAT were negatively associated with pro-inflammatory mediators and inflammatory gene expression in the serum, rumen epithelium and ileal mucosa, while being positively associated with antioxidant indices and antioxidant-/detoxification-related gene expression. Therefore, elevated circulating AG induced by GMD supplementation may be involved in the improvements in inflammation and oxidative stress in both the rumen and the ileum. However, it is important to note that these relationships are correlative and do not imply causation. Mechanistically, the protective effects of GMD may involve multiple levels of action. These effects may occur through modulation of the gastrointestinal microbiota, reduction of endotoxin burden, or direct effects of decanoic acid on epithelial inflammatory and antioxidant programs, as well as potentially through enhanced ghrelin acylation [5,14]. Taken together, our findings are consistent with the interpretation that AG may contribute to the systemic anti-inflammatory and antioxidant phenotype, while recognizing that substantial ghrelin-independent local effects of GMD may also play a meaningful role.

Transcriptomic analysis indicated that GMD suppressed several pro-inflammatory and chemotactic genes in the rumen epithelium (e.g., *IL1B*, *CXCL2*, and *GRO1*). KEGG enrichment and GSEA further revealed significant inhibition of NF-κB signaling, accompanied by coordinated downregulation of TNF, NOD-like receptor, IL-17, MAPK, and chemokine signaling pathways. This transcriptional signature closely matches the established mechanism of high-concentrate-diet-induced ruminal inflammation, wherein elevated ruminal LPS promotes excessive NF-κB activation and upregulates pro-inflammatory cytokines such as TNF-α and IL-1β, ultimately leading to inflammatory injury of the rumen epithelium [37]. In mouse and cannulated cattle studies, GMD decreased serum LPS levels and changed the gastrointestinal microbiota [5,14]. This suggests that GMD may reduce inflammation triggers by lowering endotoxin burden and improving the gut microbiome. Besides, decanoic acid itself has been reported to exert direct anti-inflammatory effects. In a pig model of intestinal dysfunction, Lee and Kang [6] showed that decanoic acid reduced TNF-α and inhibited NF-κB-related responses while improving oxidative stress and barrier-associated proteins. Additionally, GMD’s promotion of ghrelin acylation could further inhibit inflammation via ghrelin-mediated immunomodulation, which is consistent with the earlier discussion [31,32]. In contrast, the ileal transcriptomic response was relatively modest, predominantly featuring the upregulation of *GSTA1*, a key target gene in the Nrf2 regulatory network. Wang et al [38] demonstrated that decanoic acid can ameliorate cellular oxidative stress by activating the Nrf2 signaling pathway. Given that Nrf2 activity is governed by post-translational regulation (e.g., protein stabilization and nuclear translocation), unchanged upstream mRNA levels do not exclude involvement of the Nrf2 pathway in the ileum [39]. As a distal segment, the ileum may be less directly exposed to acute luminal disturbances than the rumen, which could partly explain its more modest transcriptional response [40]. Additionally, the amount of GMD reaching the distal ileum might have been insufficient to elicit a significant Nrf2-mediated response. To better elucidate the hindgut mechanism of GMD in ruminants, future studies could employ encapsulation technology to ensure targeted delivery of active components.

While the present study provides evidence supporting the beneficial effects of GMD under high-concentrate feeding conditions, several limitations should be acknowledged. This study was conducted under a high-concentrate feeding regimen without a forage-based (low-concentrate) control group. Therefore, our findings are restricted to evaluating the protective effects of GMD specifically within the context of high-concentrate feeding, for which the associated inflammatory and oxidative challenges have been well reported [1–4]. Additionally, although ghrelin-related indices were significantly correlated with improvements in inflammatory and oxidative stress parameters, these associations do not establish causality. Accordingly, future work should directly test whether the anti-inflammatory and antioxidant effects of GMD are mediated by ghrelin signaling, for example through pharmacological inhibition of GOAT or blockade of the ghrelin receptor, thereby distinguishing ghrelin-mediated effects from those exerted through direct local actions of GMD within the gastrointestinal tract.

## CONCLUSION

This study indicates that GMD supplementation may promote ghrelin acylation, alleviate systemic and gastrointestinal inflammation as well as oxidative stress in Yanbian cattle fed a high-concentrate diet. Transcriptomic analysis reveals that GMD mainly functions in the rumen by inhibiting the NF-κB signaling pathway and related pro-inflammatory genes, thereby exerting anti-inflammatory effects. In contrast, the ileal response to GMD appears more modest, characterized by upregulation of the antioxidant gene *GSTA1*. Collectively, these findings support GMD as a potential nutritional strategy for mitigating systemic and gastrointestinal inflammation and oxidative stress linked to high-concentrate diets.

## Figures and Tables

**Table 1:** Ingredient and nutrient composition of the basal diets (DM basis)

Ingredient composition	Content (% of DM)	Nutrient composition1)	Content (% of DM)
Corn straw	30.00	Dry matter	88.72
Corn meal	48.60	Crude protein	13.36
Soybean meal	13.00	Ether extract	4.83
Wheat bran	5.40	Crude ash	5.78
Sodium bicarbonate	1.00	Neutral detergent fiber	33.55
Calcium hydrogen phosphate	0.50	Acid detergent fiber	20.14
Salt	0.50	Calcium	0.74
Compound premix2)	1.00	Phosphorus	0.49
Total (%)	100.00	NEg (Mcal/kg DM)3)	1.22

1) The value reported for nutritional composition of diets was calculated based on the nutrient analysis from ingredient samples.

2) Product ingredient analysis guarantee value (/kg). Fe: 1,000 mg; Cu: 1,000 mg; Zn: 3,000 mg; Mn: 2,000 mg; I: 50 mg; Co: 10 mg; Se: 10 mg; vitamin A: 220,000 IU; vitamin D: 30,000 IU; vitamin E: 3,500 IU; vitamin K: 10 mg; vitamin H: 2 mg; ethoxyquin: 500 mg.

3) Net energy for growth (NEg) was estimated from the analyzed value of the dietary ingredients.

**Table 2:** Effects of glycerol monodecanoate (GMD) on inflammatory, antioxidant, and oxidative stress indices of serum in Yanbian Cattle

Items	Group1)		SEM	p-value

	CON	C10		
TNF-α (pg/mL)	29.71	20.77	1.898	0.016
IL-1β (pg/mL)	105.68	96.02	2.725	0.024
TAOC (U/mL)	8.57	8.99	0.105	0.044
CAT (U/mL)	45.26	53.05	1.399	0.003
SOD (U/mL)	60.64	71.20	2.969	0.075
GSH (μmol/L)	10.10	12.89	0.501	0.003
GSSG (μmol/L)	1.09	1.21	0.069	0.221
GR (U/L)	2.95	3.83	0.207	0.018
GSH-PX (U/mL)	99.09	110.48	2.654	0.029
GSH-ST (U/L)	15.66	20.73	1.147	0.015
ROS (fluorescence intensity/mL)	331.51	280.67	9.932	0.008
MDA (nmol/mL)	4.14	3.72	0.123	0.092

1) CON: basal high-concentrate diet (n = 15); C10: basal high-concentrate diet+60 g/cattle/d of GMD (n = 15).

SEM, standard error of the means; TNF-α, tumor necrosis factor-alpha; IL-1β, interleukin-1 beta; TAOC, total antioxidant capacity; CAT, catalase; SOD, superoxide dismutase; GSH, reduced glutathione; GSSG, oxidized glutathione; GR, glutathione reductase; GSH-PX, glutathione peroxidase; GSH-ST, glutathione S-transferase; ROS, reactive oxygen species; MDA, malondialdehyde.

**Table 3:** Effects of glycerol monodecanoate (GMD) on inflammatory, antioxidant, and oxidative stress indices of rumen epithelium in Yanbian Cattle

Items	Group1)		SEM	p-value

	CON	C10		
TNF-α (pg/mg protein)	12.24	8.97	0.674	0.011
IL-1β (pg/mg protein)	5.58	4.02	0.318	0.010
TAOC (U/mg protein)	1.89	2.05	0.047	0.087
CAT (U/mg protein)	17.88	19.39	0.880	0.408
SOD (U/mg protein)	21.39	23.06	0.553	0.134
GSH (μmol/g protein)	3.16	3.81	0.170	0.050
GSSG (μmol/g protein)	0.38	0.40	0.012	0.324
GR (U/g protein)	1.09	1.30	0.066	0.099
GSH-PX (U/mg protein)	42.11	42.72	1.168	0.802
GSH-ST (U/g protein)	5.48	6.04	0.228	0.248
ROS (fluorescence intensity/mg protein)	488.76	404.60	19.137	0.023
MDA (nmol/mg protein)	2.25	1.67	0.162	0.072

1) CON: basal high-concentrate diet (n = 10); C10: basal high-concentrate diet+60 g/cattle/d of GMD (n = 10).

SEM, standard error of the means; TNF-α, tumor necrosis factor-alpha; IL-1β, interleukin-1 beta; TAOC, total antioxidant capacity; CAT, catalase; SOD, superoxide dismutase; GSH, reduced glutathione; GSSG, oxidized glutathione; GR, glutathione reductase; GSH-PX, glutathione peroxidase; GSH-ST, glutathione S-transferase; ROS, reactive oxygen species; MDA, malondialdehyde.

**Table 4:** Effects of glycerol monodecanoate (GMD) on inflammatory, antioxidant, and oxidative stress indices of ileal mucosa in Yanbian Cattle

Items	Group1)		SEM	p-value

	CON	C10		
TNF-α (pg/mg protein)	7.89	6.96	0.477	0.343
IL-1β (pg/mg protein)	4.04	3.25	0.184	0.027
TAOC (U/mg protein)	1.00	1.14	0.039	0.066
CAT (U/mg protein)	17.37	18.92	0.715	0.291
SOD (U/mg protein)	17.04	18.04	0.577	0.400
GSH (μmol/g protein)	3.56	4.35	0.244	0.027
GSSG (μmol/g protein)	0.48	0.52	0.028	0.225
GR (U/g protein)	1.10	1.16	0.048	0.540
GSH-PX (U/mg protein)	30.87	34.22	1.024	0.103
GSH-ST (U/g protein)	7.19	8.80	0.410	0.043
ROS (fluorescence intensity/mg protein)	217.33	174.33	10.991	0.047
MDA (nmol/mg protein)	1.68	1.54	0.077	0.401

1) CON: basal high-concentrate diet (n = 10); C10: basal high-concentrate diet+60 g/cattle/d of GMD (n = 10).

SEM, standard error of the means; TNF-α, tumor necrosis factor-alpha; IL-1β, interleukin-1 beta; TAOC, total antioxidant capacity; CAT, catalase; SOD, superoxide dismutase; GSH, reduced glutathione; GSSG, oxidized glutathione; GR, glutathione reductase; GSH-PX, glutathione peroxidase; GSH-ST, glutathione S-transferase; ROS, reactive oxygen species; MDA, malondialdehyde.

## SUPPLEMENTARY MATERIAL

**Supplement 1**. Characteristics of gene-specific primers used for real-time quantitative polymerase chain reaction (PCR) assay.

**Supplement 2.** RNA-seq output and quality control statistics.

**Supplement 3.** Saturation simulation plot of transcriptome data.

**Supplement 4.** List of differentially expressed genes in the transcriptome of rumen epithelium and ileal mucosa.
