# Supplementary material for: Effects of dietary glycerol monodecanoate supplementation on inflammation, oxidative stress, ghrelin acylation, and gastrointestinal transcriptome in Yanbian cattle fed a high-concentrate diet
Source: Anim Biosci. 2026 Apr 2;39(9):251032. doi: 10.5713/ab.251032 (PMC13547627; doi:10.5713/ab.251032)
Supplement: Supplementary file 1 [file ab-251032-Supplementary-1.pdf]

**Supplement 1**. Characteristics of gene-specific primers used for real-time quantitative polymerase chain reaction (PCR) assay

| Gene[1] | NCBI GenBank accession number | Sequences (forward/reverse) (5′–3′) | Amplicon size (bp) |
|---|---|---|---|
| *GHRL* | NM_174067.2 | F: GACGAGCTGGAAATCCGGT<br>R: TCGTTAGCCAGGGTTTCTTCAG | 143 |
| *MBOAT4* | NM_001192257.3 | F: GCCGACCTTCCTCTTTCAGAT<br>R: AGAGAGTGATGCAGAACCTTG | 110 |
| *TLR4* | NM_174198.6 | F: TGAGACATTTAGGCAGCCATAACTTC<br>R: CCGTCAGTATCAAGGTGGAGAGG | 135 |
| *MYD88* | NM_001014382.2 | F: CTCATTGAGAAGAGGTGCCGT<br>R: ATCAGTCGCTTCTGATGGGC | 128 |
| *RELA* | NM_001080242.2 | F: CGCTTCCGCTACAAGTGTGAG<br>R: GGGTGGGTCTTGGTGGTGTC | 80 |
| *TNF* | NM_173966.3 | F: CAACGGTGTGAAGCTGGAAGAC<br>R: TGAAGAGGACCTGTGAGTAGATGAG | 80 |
| *IL1B* | NM_174093.1 | F: GCTCTCCACCTCCTCTCACAG<br>R: ATCTTGTTGTCTCTTTCCTCTCCTTG | 86 |
| *NFE2L2* | NM_001011678.2 | F: TTTGGCAGAGACATTCCCGTTTG<br>R: CCTGAGGAGGAGCAGTGAAGAC | 119 |

| | | | |
|---|---|---|---|
| *KEAP1* | NM_001101142.1 | F: TGTCCTCAACCGTCTGCTCTAC<br>R: TCCGCCACTCGTTTCTCTCTG | 98 |
| *HMOX1* | NM_001014912.1 | F: GCCAGTGCCACCAAGTTCAAG<br>R: TGAGCAGGAAGGCGGTCTTG | 112 |
| *NQO1* | NM_001034535.1 | F: ATGAAGGAGGCTGCCATAGAGG<br>R: CTGGAGATGACGGGATTGAAGTTC | 95 |
| *GSTA1* | NM_001078149.1 | F: TTCCCTCTGCTAAAGGCCCT<br>R: CTTCCTCTGGCTGCCAGG | 84 |
| *ACTB* | NM_173979.3 | F: AGGCATCCTGACCCTCAAGTA<br>R: GCTCGTTGTAGAAGGTGTGGT | 95 |
| *GAPDH* | NM_001034034.2 | F: ACGGCACAGTCAAGGCAGAG<br>R: CACATACTCAGCACCAGCATCAC | 116 |

[1] Gene abbreviations: *GHRL* (ghrelin); *MBOAT4* (ghrelin O-acyltransferase); *TLR4* (Toll-like receptor 4); *MYD88* (myeloid differentiation primary response 88); *RELA* (NF-κB p65 subunit); *TNF* (tumor necrosis factor); *IL1B* (interleukin 1 beta); *NFE2L2* (nuclear factor erythroid 2–related factor 2); *KEAP1* (Kelch-like ECH-associated protein 1); *HMOX1* (heme oxygenase 1); *NQO1* (NAD(P)H quinone dehydrogenase 1); *GSTA1* (glutathione S-transferase alpha 1); *ACTB* (beta-actin); *GAPDH* (glyceraldehyde-3-phosphate dehydrogenase).
