# Supplementary material for: Effects of dietary glycerol monodecanoate supplementation on inflammation, oxidative stress, ghrelin acylation, and gastrointestinal transcriptome in Yanbian cattle fed a high-concentrate diet
Source: Anim Biosci. 2026 Apr 2;39(9):251032. doi: 10.5713/ab.251032 (PMC13547627; doi:10.5713/ab.251032)
Supplement: Supplementary file 2 [file ab-251032-Supplementary-2.pdf]

Supplement 2. RNA-seq output and quality control statistics

Samples[1]	Clean reads[2]	Clean bases[3]	GC Content (%)[4]	%≥Q30[5]
Rumen epithelium				
CON-1	20,887,441	6,230,270,821	47.94	94.80
CON-2	24,150,352	7,208,368,250	47.60	95.62
CON-3	22,977,307	6,852,371,333	47.54	95.12
CON-4	19,912,712	5,950,782,793	48.64	95.63
CON-5	20,997,629	6,269,381,797	47.88	95.72
CON-6	21,441,798	6,401,200,794	50.31	95.11
C10-1	19,882,534	5,925,335,530	48.17	95.68
C10-2	22,150,192	6,616,870,048	46.71	96.16
C10-3	23,055,196	6,886,804,572	49.03	95.78
C10-4	20,204,295	6,027,567,377	46.45	94.94
C10-5	20,614,168	6,151,293,320	46.37	96.00
C10-6	21,232,513	6,341,013,593	47.51	95.95
Ileal mucosa				
CON-1	19,699,592	5,890,969,428	50.53	95.41
CON-2	20,297,281	6,070,726,806	49.67	95.51
CON-3	19,799,569	5,920,680,828	50.13	95.43
CON-4	20,844,080	6,227,942,691	49.58	95.43
CON-5	21,944,567	6,559,316,908	49.28	95.78
CON-6	19,843,700	5,929,638,730	48.02	95.40

C10-1	20,506,822	6,132,909,988	50.96	95.63
C10-2	20,589,315	6,149,316,771	47.52	95.68
C10-3	21,002,876	6,278,751,430	49.35	95.56
C10-4	20,763,105	6,202,512,245	49.51	95.49
C10-5	19,889,519	5,941,150,215	49.30	95.37
C10-6	21,076,505	6,294,501,059	49.51	95.51

[1] CON: basal high-concentrate diet; C10: basal high-concentrate diet + 60 g/cattle/day GMD.

[2] Clean reads are the total number of paired-end reads in clean data.

[3] Clean bases are the total number of bases in the clean data.

[4] GC content (%) is the percentage of G and C bases among all bases in the clean data.

[5] ≥Q30 (%) is the percentage of bases with a Phred quality score ≥ 30 in the clean data.
