# Supplementary material for: Effects of dietary glycerol monodecanoate supplementation on inflammation, oxidative stress, ghrelin acylation, and gastrointestinal transcriptome in Yanbian cattle fed a high-concentrate diet
Source: Anim Biosci. 2026 Apr 2;39(9):251032. doi: 10.5713/ab.251032 (PMC13547627; doi:10.5713/ab.251032)
Supplement: Supplementary file 3 [file ab-251032-Supplementary-3.pdf]

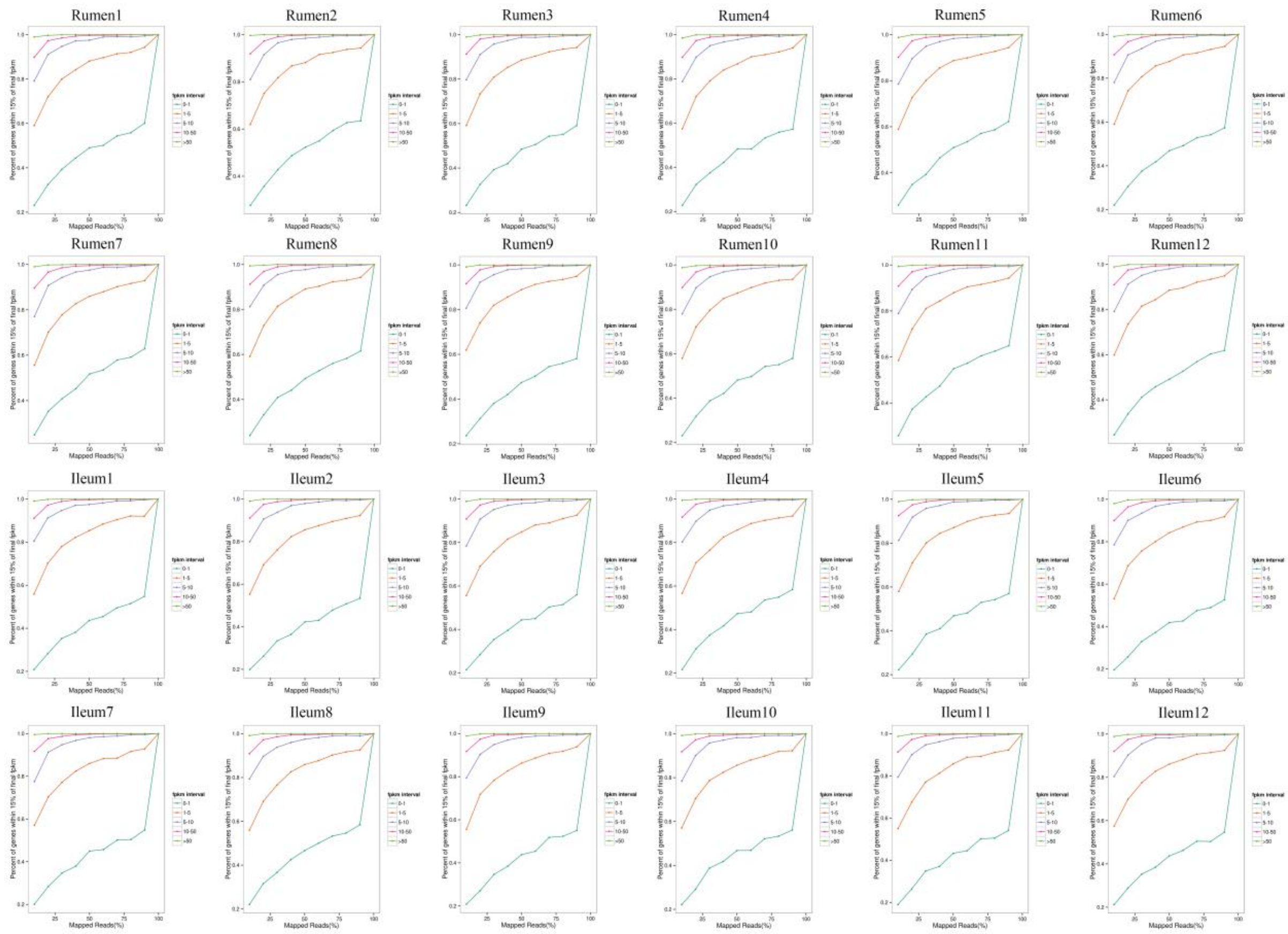

Supplement 3. Saturation simulation plot of transcriptome data. The horizontal axis represents the percentage of sampled reads mapped to the genome relative to the total mapped reads, and the vertical axis represents the percentage of genes with expression differences less than 15% across all sampling results within each FPKM interval. Numbers 1–6 represent the CON group, and numbers 7–12 represent the C10 group.
