# Supplementary material for: Effects of dietary glycerol monodecanoate supplementation on inflammation, oxidative stress, ghrelin acylation, and gastrointestinal transcriptome in Yanbian cattle fed a high-concentrate diet
Source: Anim Biosci. 2026 Apr 2;39(9):251032. doi: 10.5713/ab.251032 (PMC13547627; doi:10.5713/ab.251032)
Supplement: Supplementary file 4 [file ab-251032-Supplementary-4.pdf]

Supplement 4. List of differentially expressed genes in the transcriptome of rumen epithelium and ileal mucosa

Gene	FDR[1]	log_2FC[2]	CON vs C10 regulated[3]
Rumen			
ALPL	0.049	-0.87	down
CCR10	0.018	-2.56	down
CSF3R	0.020	-1.71	down
CXCL2	0.008	-1.93	down
CXCR2	0.009	-1.73	down
DUSP2	0.024	-1.53	down
ELF3	0.001	-1.05	down
FCMR	0.095	-1.71	down
GRO1	0.024	-1.86	down
HSPA6	0.095	-1.58	down
HYOU1	0.077	-0.91	down
IL1B	0.009	-2.01	down
ITGAL	0.002	-1.88	down
MEFV	0.024	-2.06	down
MZB1	0.024	-1.90	down
NAIP	0.011	-1.36	down
PLA2G4D	0.020	-0.99	down

PLA2G4E	0.024	-0.84	down
PRSS27	0.006	-1.52	down
RGS5	0.098	0.63	up
SLC5A1	0.095	-1.37	down
SPNS2	0.008	-0.63	down
STARD9	0.053	0.61	up
THEMIS2	0.015	-1.47	down
TRPV3	0.001	-0.84	down
VAV1	< 0.001	-1.52	down
ZBED2	0.024	-1.01	down
Ileum			
AAMDC	0.081	0.50	up
GSTA1	0.040	2.16	up
LOC132344467	0.041	3.61	up
LOC508858	0.005	6.00	up
MS4A18	0.074	-1.89	down

[1] FDR: false discovery rate.

[2] log_2FC: Log2 fold change

[3] CON vs C10 regulated: Regulation direction. “Up” indicates higher expression in the C10 group compared to the CON group; “down” indicates lower expression in the C10 group.
